# Badnaviruses and banana genomes: a long association sheds light on *Musa* phylogeny and origin

**DOI:** 10.1111/mpp.13019

**Published:** 2020-11-24

**Authors:** Matthieu Chabannes, Marc Gabriel, Abderrahmane Aksa, Serge Galzi, Jean‐François Dufayard, Marie‐Line Iskra‐Caruana, Emmanuelle Muller

**Affiliations:** ^1^ CIRAD, UMR BGPI, University of Montpellier, Montpellier SupAgro Montpellier France; ^2^ CIRAD, UMR AGAP, University of Montpellier, CIRAD, INRA, Montpellier SupAgro Montpellier France

**Keywords:** *Badnavirus*, banana streak virus, endogenous pararetrovirus, host and virus coevolution, *Musa* phylogeny

## Abstract

Badnaviruses are double‐stranded DNA pararetroviruses of the family *Caulimoviridae*. Badnaviral sequences found in banana are distributed over three main clades of the genus *Badnavirus* and exhibit wide genetic diversity. Interestingly, the nuclear genome of many plants, including banana, is invaded by numerous badnaviral sequences although badnaviruses do not require an integration step to replicate, unlike animal retroviruses. Here, we confirm that banana streak viruses (BSVs) are restricted to clades 1 and 3. We also show that only BSVs from clade 3 encompassing East African viral species are not integrated into *Musa* genomes, unlike BSVs from clade 1. Finally, we demonstrate that sequences from clade 2 are definitively integrated into *Musa* genomes with no evidence of episomal counterparts; all are phylogenetically distant from BSVs known to date. Using different molecular approaches, we dissected the coevolution between badnaviral sequences of clade 2 and banana by comparing badnavirus integration patterns across a banana sampling representing major *Musa* speciation events. Our data suggest that primary viral integrations occurred millions of years ago in banana genomes under different possible scenarios. Endogenous badnaviral sequences can be used as powerful markers to better characterize the *Musa* phylogeny, narrowing down the likely geographical origin of the *Musa* ancestor.

## Graphical abstract

Banana‐related badnavirus sequences have undergone different coevolution processes with their host plant over the time, with some of them being either integrated or episomal while others are both integrated and episomal.

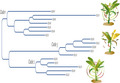

## 1 INTRODUCTION

Although integration is not a requisite in the life cycle of pararetroviruses, integrated pararetrovirus sequences do occur in the nuclear genomes of several plant species, including banana (Harper et al., 1999; Lockhart & Jones, 2000; Ndowora et al., 1999), citrus (Yu et al., 2019), dahlia (Pahalawatta et al., 2008), fig (Laney et al., 2012), grape (Bertsch et al., 2009), petunia (Richert‐Poggeler et al., 2003), pineapple (Gambley et al., 2008), potato (Hansen et al., 2005), rice (Kunii et al., 2004), tobacco, and tomato (Gregor et al., 2004; Jakowitsch et al., 1999; Staginnus et al., 2007). Integration into host chromosomes probably occurs in the nucleus (Hull, 2002) via nonhomologous end‐joining (NHEJ) during repair of host DNA breaks, as reported for several endogenous viral elements (EVEs) (Feschotte & Gilbert, 2012; Holmes, 2011). These integrated sequences are relics of quite ancient infection events, so their presence is not necessarily associated with current infection, especially when present only as partial viral sequences or nonfunctional sequences with null mutations.

Usually, pararetroviral integrations have no deleterious impact on their host plants because they are untranslatable sequences. However, in some cases, integrated sequences contain a functional full‐length viral genome that can be activated, leading to systemic infection of the host plant. Also known as infective integration, examples include *Petunia vein clearing virus* (genus *Petuvirus*) in petunia (Richert‐Poggeler & Shepherd, 1997), *Tobacco vein clearing virus* (genus *Solendovirus*) in tobacco (Gregor et al., 2004), and banana streak viruses (BSVs; genus *Badnavirus*) in banana (Gayral et al., 2008; Harper et al., 1999; Ndowora et al., 1999). BSVs in banana are by far the most economically significant examples; indeed, banana—one of the oldest domesticated crops in the world—is ranked as the world's sixth most important food crop in terms of gross production value after cassava, potato, rice, wheat, and maize (FAOStat, 2014), and first among fruit crops.

Most modern banana cultivars arose via traditional selection processes (Perrier et al., 2011). The seedy progenitors of all domesticated banana cultivars are *Musa acuminata* (A genome) and *Musa balbisiana* (B genome) and, to a much lesser extent, *Musa schizocarpa* (S genome) and *Musa textilis*/*Musa maclayi* (T genome) (Carreel et al., 2002; Daniells et al., 2001a). *M. acuminata* exhibits large diversity based on morphological and molecular characters, and up to nine different subspecies are known (Christelova et al., 2011; Daniells et al., 2001a). *M. balbisiana* shows comparatively narrower diversity, with a more restricted centre of origin (Perrier et al., 2011). Interestingly, infective endogenous BSV sequences (eBSV)—found exclusively in the *M. balbisiana* B genome to date—belong to three distinct BSV species: *Banana streak GF virus*, *Banana streak IM virus*, and *Banana streak OL virus* (Chabannes et al., 2013; Gayral et al., 2008; Iskra‐Caruana et al., 2010). Several reports have noted the presence of partial badnaviral sequences also in *M. acuminata* and/or *M. balbisiana* genomes (Geering et al., 2001; Ndowora et al., 1999), but most significant was the description of 33 distinct groups of banana endogenous viruses (BEV) related to either *M. acuminata* or *M. balbisiana* genomes (Geering et al., 2005).

**FIGURE 1:**
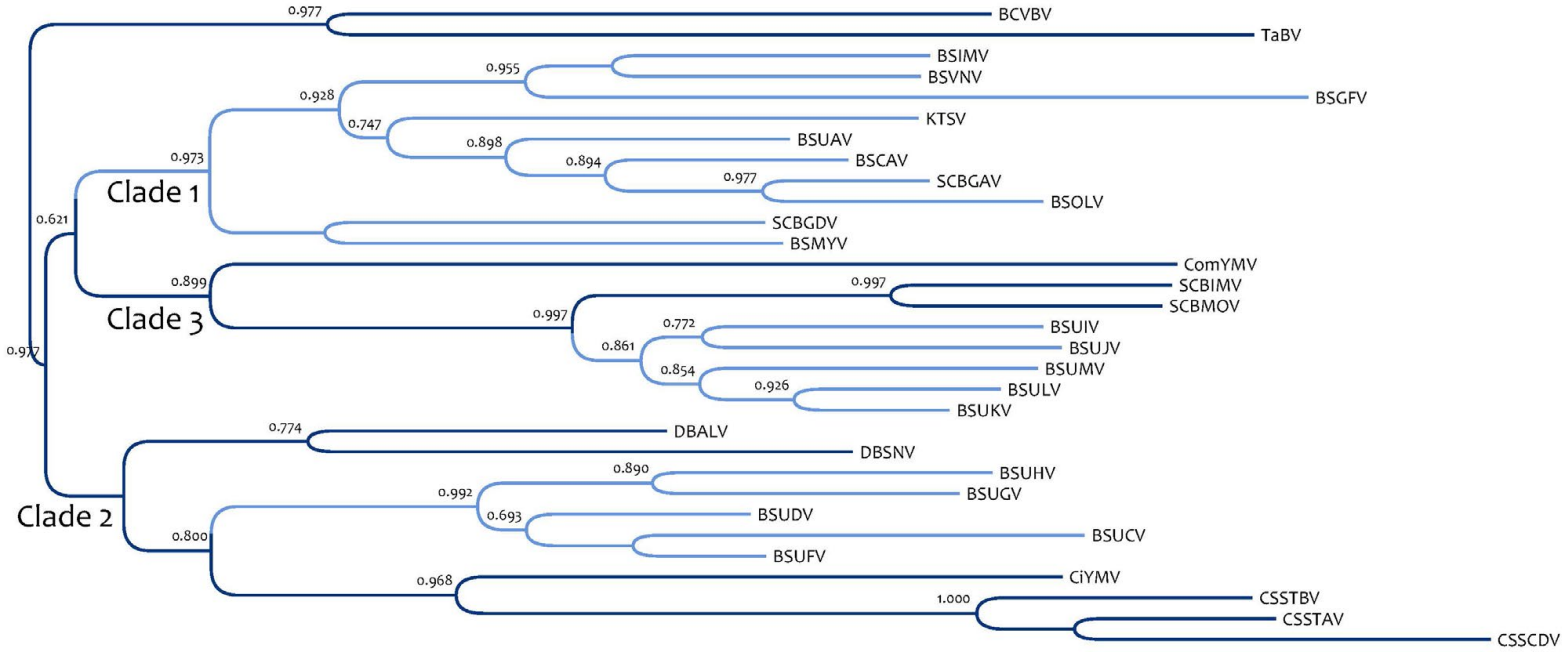
Maximum‐likelihood phylogeny based on RTase/RNase H region. Statistical aLRT SH‐like branch supports given above nodes when >0.6. Virus or sequences names (GenBank numbers): BSUCV (AJ968464), BSUDV (AJ968465), BSUFV (AJ968469), BSUGV (AJ968470), BSUHV (AJ968472), Banana streak Cavendish virus (BSCAV, HQ593111), *Banana streak MY virus* (BSMYV, AY805074), *Banana streak GF virus* (BSGFV, AY493509), *Banana streak IM virus* (BSIMV, HQ659760), *Banana streak OL virus* (BSOLV, AJ002234), *Banana streak VN virus* (BSVNV, AY750155), *Banana streak UA virus* (BSUAV, HQ593107), *Banana streak UI virus* (BSUIV, HQ593108), Banana streak UJ virus (BSUJV, AJ968501), Banana streak UK virus (BSUKV, AJ968504), *Banana streak UL virus* (BSULV, HQ593109), *Banana streak UM virus* (BSUMV, HQ593110), *Bougainvillea chlorotic vein banding virus* (BCVBV, EU034539), *Cacao swollen shoot Togo B virus* (CSSTBV, L14546), *Cacao swollen shoot CD virus* (CSSCDV, JN606110), *Cacao swollen shoot Togo A virus* (CSSTAV, AJ781003), *Citrus yellow mosaic virus* (CiYMV, AF347695), *Commelina yellow mottle virus* (ComYMV, X52938), *Dioscorea bacilliform AL virus* (DBALV, X94576‐581), *Dioscorea bacilliform SN virus* (DBSNV, DQ822073), *Kalanchoe top‐spotting virus* (KTSV, AY180137), *Sugarcane bacilliform IM virus* (SCBIMV, AJ277091), *Sugarcane bacilliform MO virus* (SCBMOV, M89923), *Sugarcane bacilliform Guadeloupe A virus* (SCBGAV, FJ824813), *Sugarcane bacilliform Guadeloupe D virus* (SCBGDV, FJ439817), *Taro bacilliform virus* (TaBV, AF357836)

The genomes of badnaviruses contain three main open reading frames (ORFs), with the largest encoding a movement protein, a capsid protein, an aspartic protease, a reverse transcriptase (RTase), and a ribonuclease H (RNAse H). Badnavirus genetic diversity (based on partial sequences of the RTase and RNase H genes) in banana appears large and complex, as viral sequences generated to date are distributed over three different clades within the diversity of the genus *Badnavirus* (Gayral & Iskra‐Caruana, 2009; Harper et al., 2005) (Figure 1). Importantly, the 11 full‐length sequenced episomal BSV species responsible for banana streak disease described to date belong only to clades 1 and 3; clade 3 encompasses East African BSV species exclusively (Geering et al., 2005, 2011; Harper & Hull, 1998; James et al., 2011). All other (partial) badnavirus sequences described in banana are in clade 2. However, controversially, Harper et al. (2005) associated banana streak disease in Uganda (banana genotype East African Highland [EAH] AAA) with badnaviral sequences belonging to clade 2. With the previous International Committee on Taxonomy of Viruses (ICTV) demarcation criteria of an 85% nucleotide divergence threshold (now fixed at 80%), they proposed five novel BSV Uganda BSUXV species (X = species descriptor): BSUCV, BSUDV, BSUFV, BSUGV, and BSUHV. Subsequently, examining different evolutionary parameters of episomal and endogenous sequences, Gayral and Iskra‐Caruana (2009) suggested that BSV sequences from clade 2, including the five BSUXV species, exist as endogenous sequences only, unlike BSV sequences from clade 3, which are episomal.

Here, we focused our research on banana badnaviral clade 2 sequences to categorize their exact nature (integrated and/or episomal) and estimate their distribution in the family Musaceae. Performing Southern blot and immunocapture PCR (IC‐PCR) tests on a wide sample survey of 109 samples of EAH AAA banana collected in Uganda, we extended sampling to include diploid banana plants representative of the diversity of the family Musaceae. Interestingly, the different BEV integration patterns observed indicate that banana genomes have been colonized extensively over time under different possible scenarios. BEVs could thus serve as phylogenetic markers to precisely map *Musa* phylogeny. This coevolution between clade 2 badnaviral sequences and banana allows us to better estimate the age of different integration events and narrow the likely geographical origin of the *Musa* ancestor.

## 2 RESULTS

### 2.1 Detection of BSV sequences in Ugandan EAH AAA banana samples

We estimated the distribution and prevalence of BSV species in 109 samples representative of local banana diversity collected from EAH AAA bananas in Uganda. All samples were first subjected to DNase I‐treated IC‐PCR using specific primers (Table 1) to detect episomal forms of the main circulating BSVs, banana streak OL virus (BSOLV), banana streak GF virus (BSGFV), banana streak IM virus (BSIMV), and banana streak MY virus (BSMYV), belonging to species of clade 1. Among 91 EAH AAA plant samples exhibiting typical disease symptoms, only 34 (c.1:3) were infected by the BSVs from clade 1 tested here: 32 plants by BSOLV and two by BSIMV (Table 2). Symptomless plants were also infected, one by BSOLV and four by BSIMV. In total, 33/109 plants were infected with BSOLV and six with BSIMV, with only one plant coinfected with both viruses. No samples were infected with either BSMYV or BSGFV.

**TABLE 1:** PCR primers used to detect badnaviral species of the different clades in banana plants

Specificity	Forward primer (5′–3′)	Reverse primer (5′–3′)	Tm (°C)	Number of PCR cycles	Amplicon size (bp)
IC‐PCR for species of clade 1					
BSOLV	ATCTGAAGGTGTGTTGATCAATGC	GCTCACTCCGCATCTTATCAGTC	62	30	522
BSGFV	ACGAACTATCACGACTTGTTCAAGC	TCGGTGGAATAGTCCTGAGTCTTC	62	30	475
BSIMV	TGCCAACGAATACTACATCAAC	CACCCAGACTTTTCTTTCTAGC	62	25	383
BSMYV	TAAAAGCACAGCTCAGAACAAACC	CTCCGTGATTTCTTCGTGGTC	62	30	588
PCR for species of clade 2					
BEV UC	CTGAAGAATGCACCAGCAATATTC	TCCATACATCCGTCAGTTTCGAG	60	35	542
BEV UD	GGTACAGAACAATTYATTGCTGTGTAC	CCAGTTGGGCTTGTTTTTGAATAC	60	35	362
BEV UF	ACTGCTTCAAAGGTACGGAACAAT	CTAGATCCGGGAGATTTTGTACCA	60	35	450
BEV UG	TGGACGACTGCTTTCGAGGT	CATACATCCGTCCACCTCTAGGA	60	35	506
BEV UH	GCATCTTCATAAAATGCTAGAAATCTGT	TCTGGGGGTGGTACTTCTAGATCA	60	35	381
BEV NGA	CCGTGTTCCAAAGGAAAATGGAC	CATGCATCCGTCTGTTTCAAGTATTATG	60	35	524
BEV P	CAAAGGTACAGAACAATTTATAGCAGTC	GGAGTAAAGTGGTCCTAACAGCCTT	60	35	348
BEV Q	CCTGGTATTCTCAGAGAATGAAGAA	GTCTTCGGGGGGAACTTCTAAG	60	35	418
PCR for species of clade 3					
BSUIV	AAGAAGARCATGCGGAGCATTT	GCATCCTCTGGGGGTATTGC	55	35	401
BSUJV	CAGAAGCCTTTATAGCAGTTTACATT	TCCTGCTTCATCCTTCTGATAATT	55	35	412
BSUKV	GATGATATTCTGGTTTTTTCAGAAACTA	CAGTTTCAATTACTATGTATGCATCTGA	55	35	448
BSULV	TGGTTTTCTCAGAAACTGARGAAGA	ATGTATGCATCTTGAGGGGGTAT	53	35	424
BSUMV	AGGGACTGAGGCATTCATAGC	TTCATYCTTCTGATTATCTTCCA	50	35	412

**TABLE 2:** Badnavirus diversity in EAH AAA banana samples from Uganda

Banana samples	Clade 1				Clade 2	Clade 3				
	BSOLV	BSIMV	BSMYV	BSGFV	BSUCV, BSUDV, BSUFV, BSUGV, BSUHV	BSUIV	BSUJV	BSUKL	BSULV	BSUMV
With typical BSV symptoms (91)	32	2	0	0	91	20	2	0	34	34
Symptomless or doubtful symptoms (18)	1	4	0	0	18	2	0	0	0	0

*Note*. Numbers refer to the number of plants.

EAH AAA banana genetic diversity encompasses the five following clone sets: Nakitembe, Musakala, Nakabulu, Nfuuka, and Nbide (beer cultivar).

Because the polyclonal antiserum used for IC‐PCR did not reliably detect BSVs from clade 3, every sample was subjected to direct PCR using specific primers (Table 1) to detect banana streak UI virus (BSUIV), banana streak UJ virus (BSUJV), banana streak UK virus (BSUKV), banana streak UM virus (BSUMV), and banana streak UL virus (BSULV). Of 91 plants with symptoms, 82 appeared infected by at least one of the five BSVs tested from clade 3. No samples were infected by BSUKV. Of the four other BSVs in this clade, BSUJV is strongly under‐represented in our samples, with only two plants harbouring BSUJV sequences compared to 20, 34, and 34 for BSUIV, BSUMV, and BSULV, respectively (Table 2). Two symptomless banana plants were infected with BSVs from clade 3, both carrying BSUIV. The finding that BSVs from clade 3 are detected in most plants with symptoms, and absent from most symptomless EAH AAA plants, supports the episomal nature of viruses in these five BSV species.

Similarly, direct PCR using specific primers identified BSV sequences from clade 2. All samples, regardless of symptoms, contained all five BSV sequences belonging to clade 2 (Table 2). Their systematic presence in every EAH AAA banana tested suggests that sequences from all five species are probably integrated into the *M. acuminata* genome.

### 2.2 Are badnaviral sequences from clades 2 and 3 integrated in Ugandan EAH AAA genotypes?

**FIGURE 2:**
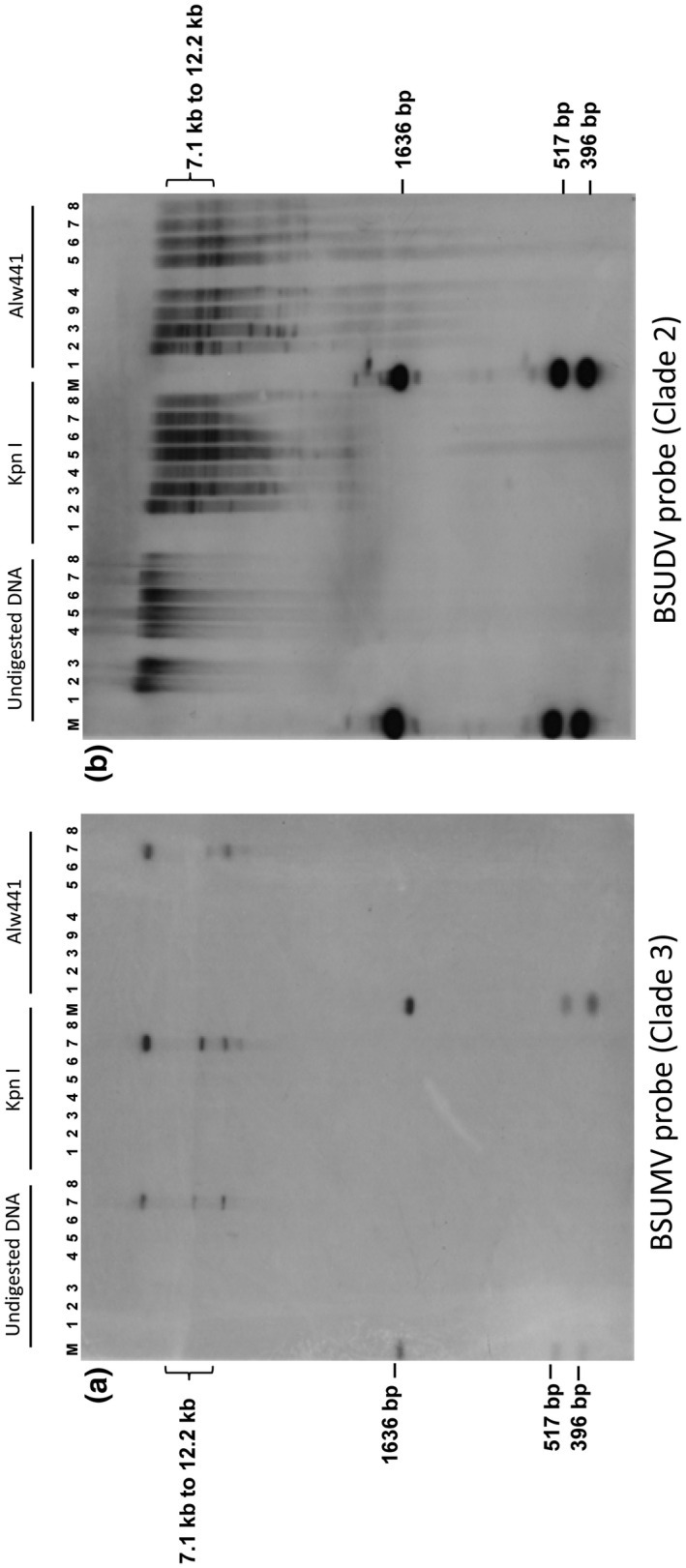
Southern blot hybridization of genomic DNA from EAH AAA banana plants using clade 3 (BSUMV; a) and clade 2 (BSUDV; b) badnaviral sequences as probes. Total genomic DNA (undigested, *Kpn*I‐digested, *Alw*441‐digested): 1, Pisang Klutuk Wulung (PKW) (BB genotype); 2, Cavendish (CAV) (AAA genotype); 3, Pahang (AA genotype); 4–9, EAH AAA plants (4, 6, 9, healthy samples [symptomless and no bacilliform particles]; 5, BSUIV‐infected; 7, BSUMV‐infected; 8, BSUIV and BSULV coinfected). M, 1 kb ladder from Invitrogen

**FIGURE 3:**
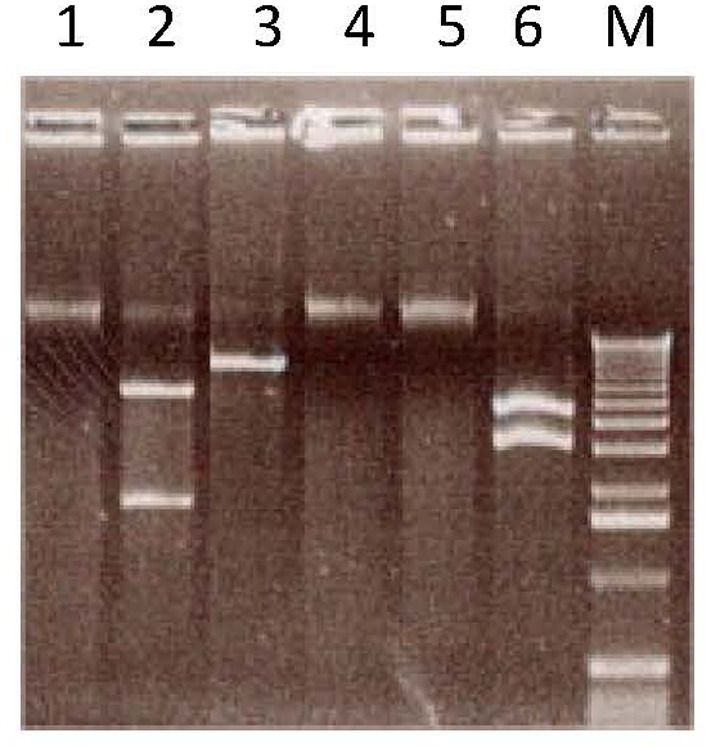
Agarose gel analysis of rolling circle‐amplified (RCA) DNA. Undigested (lanes 1, 4, and 5), *Kpn*I‐digested (lane 2), *Bam*HI‐digested (lane 3), and *Pst*I‐digested (lane 6) RCA products derived from BSUIV (lanes 1 and 2) and BSUMV (lanes 3, 4, 5, and 6) infected banana plants. M, 1 kb ladder from Invitrogen

To establish whether badnavirus sequences corresponding to species from clades 2 and 3 are episomal, integrated or both, Southern blot analyses were performed. Probes corresponding to BSUDV and BSUFV (clade 2), and BSUIV, BSULV, and BSUMV (clade 3) were hybridized to undigested and digested genomic DNA of three banana samples with symptoms and three samples without symptoms (Figures 2, S1, and S2) representative of the whole EAH AAA diversity. Figure 2 illustrates results obtained with BSUDV and BSUMV probes. The three samples with symptoms were negative for BSVs from clade 1, one was infected with BSUIV (sample 5), another with BSUMV (sample 7), and the third was coinfected with BSULV and BSUIV (sample 8). As expected, hybridization with the BSUMV probe (Figure 2a) yielded a signal only with sample 7, testing positive with BSUMV primers. The upper band corresponds either to viral concatemers, as described by Geijskes et al. (2004), or, more probably, to episomal viral DNA present in the heavy plant genomic DNA, with the two lower bands corresponding to open circular and supercoiled viral double‐stranded (ds)DNA (the lowest band). The viral sequence (which was not available when the experiments were carried out) does not harbour a recognition sequence for either *Kpn*I or *Alw*441; the hybridization pattern was, therefore, similar to that of undigested DNA (Figure 2a). Similar patterns were observed with BSUIV and BSULV probes in samples testing positive with the corresponding primers (Figures S1 and S2). Thus, the three BSV species belonging to clade 3 do not seem to be integrated in the samples tested, representing the five clone sets of the EAH AAA genotypes, or in Cavendish (AAA), Pahang (AA), and PKW (BB) control plants. Additionally, rolling circle amplification (RCA) performed on one BSUIV and two BSUMV infected banana samples gave products of high molecular weight in all cases. RCA products were digested and the sum of the fragments obtained was in each case of the order of 7.5 kb (Figure 3). For one of the samples, the fragments were cloned and sequenced, confirming the presence of BSUMV (data not shown).

In contrast, and corroborating PCR results, the BSUDV probe hybridized with all (digested and undigested) *M. acuminata* samples tested, including virus‐free banana plants cultivars Cavendish AAA and Pahang AA (Figure 2b). We also observed high molecular weight signals on undigested DNA (>15 kb) without the two lower bands (open circular/supercoiled viral dsDNA, cf. Figure 2a) associated with multiple band patterns; the cumulative size of all bands was >7 kb per sample on digested DNA. This result indicates that BSUDV is integrated into the genomes of all genotypes tested except PKW. Similar patterns were observed with a BSUFV probe (Figure S1). We also performed a parallel immunosorbent electron microscopy experiment on eight symptomless banana samples containing only clade 2 sequences (also analysed in the Southern blot experiment) and on nine samples with symptoms containing clade 3 and clade 2 sequences (including samples 7 and 8 in Figure 2, and sample 9 in Figure S1). As expected, viral particles were observed only for the samples with symptoms containing clade 3 sequences (Figure S3).

Consequently, and given the absence of corresponding episomal virus in the samples tested so far and identity with any known viral species far below the threshold of 80%, we definitively conclude that clade 2 BSUDV and BSUFV in our tested banana samples are exclusively endogenous badnaviral‐related sequences. According to the PCR results (Table 2), we can extend this statement to other clade 2 species and reclassify these BSUXV species under the general term BEV UX, as defined by Geering et al. (2005).

### 2.3 Are clade 2 badnaviral sequences present in the *Musa* diversity?

To gain a better overview of the presence/spread of clade 2 badnaviral sequences in banana genomes over evolutionary time, we looked for clade 2 BEVs in diploid banana plants representing subspecies of *M. acuminata* and *M. balbisiana*, as well as other species from the genus *Musa* and the two other genera of Musaceae (*Ensete* and *Musella*).

We first analysed diversity of clade 2 badnaviral sequences using PCR primers specific for different clade 2 BEVs (Table 1). BEV‐NGA corresponds to a distinct BEV integrated in the genome of *M. acuminata* subsp. *malaccensis* ‘Pahang’ (D’Hont et al., 2012). BEVs UC and UF generated amplification products from all *M. acuminata* samples (AA1–AA14 and AA26) and all *M. balbisiana* samples (BB15–BB20) (Table 3). BEV UG primers gave the same results, with the exception of four *M. acuminata* samples (AA4, AA5, AA14, and AA26), where no amplification was obtained. Primers specific to BEVs UD, UH, and NGA amplified products from all *M. acuminata* samples. In contrast, no amplification was obtained for the five *M. balbisiana* samples. Other species of the family Musaceae yielded very diverse results. Interestingly, *M. laterita* (M22) contained all six BEVs tested, *M. ornata* (M25) five BEVs (only BEV UD missing), *M. basjoo* (M24) and *M. itinerans* (M27) harboured both BEV UC and BEV UG, and BEV UH was detected in *M. schizocarpa* (M21) (Table 3). Furthermore, no BEVs from clade 2 were amplified in *M. textilis* (M23) and *M. coccinea* (M28), or from samples of the other two Musaceae genera (M32 and M34) (Table 3).

**TABLE 3:** PCR amplification results

Species	Plant samples	BEV‐specific primers							
		BEV UC	BEV UD	BEV UF	BEV UG	BEV UH	BEV NGA	BEV P	BEV Q
*M. acuminata*	AA1/*errans*	+	+	+	+	+	+	−	−
	AA2/*zebrina*	+	+	+	+	+	+	−	−
	AA3/*siamea*	+	+	+	+	+	+	−	−
	AA4/*malaccensis*	+	+	+	−	+	+	−	−
	AA5/*malaccensis*	+	+	+	−	+	+	−	−
	AA6/*truncata*	+	+	+	+	+	+	−	−
	AA7/*burmannica*	+	+	+	+	+	+	−	−
	AA8/*microcarpa*	+	+	+	+	+	+	−	−
	AA9/*malaccensis* (ITC0250)	+	+	+	+	+	+	−	−
	AA10/*banksii*	+	+	+	+	+	+	−	−
	AA11/*siamea*	+	+	+	+	+	+	−	−
	AA12/*malaccensis* (ITC0399)	+	+	+	+	+	+	−	−
	AA13/*burmannicoides*	+	+	+	+	+	+	−	−
	AA14/*siamea*	+	+	+	−	+	+	−	−
	AA26/*banksii*	+	+	+	−	+	+	−	−
*M. balbisiana*	BB15/Honduras	+	−	+	+	−	−	+	+
	BB16/Cameroun	+	−	+	+	−	−	+	+
	BB17/Pisang Klutuk Wulung	+	−	+	+	−	−	+	+
	BB18/Butuhan	+	−	+	+	−	−	+	+
	BB19/Lal Velchi	+	−	+	+	−	−	+	+
	BB20/Papouasie Nouvelle Guinée	+	−	+	+	−	−	+	+
Other species of the family Musaceae	M21/*M*. *schizocarpa*	−	−	−	−	+	−	−	−
	M22/*M. laterita*	+	+	+	+	+	+	−	−
	M23/*M. textilis*	−	−	−	−	−	−	−	−
	M24/*M. basjoo*	+	−	−	+	−	−	−	−
	M25/*M. ornata*	+	−	+	+	+/−	+	−	−
	M27/*M. itinerans*	+	−	−	+	−	−	−	−
	M28/*M. coccinea*	−	−	−	−	−	−	−	−
	M32/*Ensete ventricosum*	−	−	−	−	−	−	−	−
	M34/*Musella lasiocarpa*	−	−	−	−	−	−	−	−

*Note*. AA diploid *M. acuminata* bananas, BB diploid *M. balbisiana* bananas, and other species from the family Musaceae.

**FIGURE 4:**
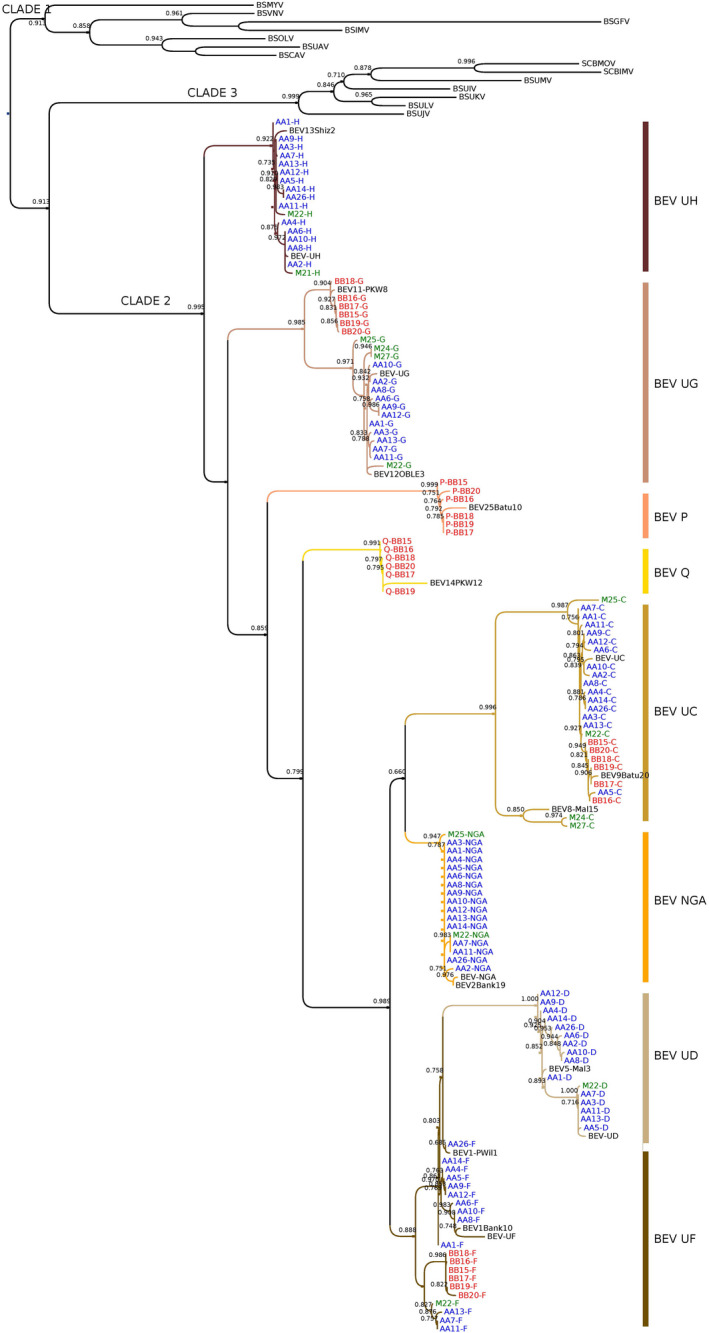
Maximum‐likelihood phylogeny of clade 2 badnavirus sequences based on RTase/RNase H region. Statistical aLRT SH‐like branch supports given above nodes when >0.6. BEV sequences (Geering et al., 2005) close to our BEV groups are included. BSV species from clades 1 and 3 are used as outgroups. AA1–M27 refer to sample number. Samples: blue, AA; red, BB; green, M. Sequences used for comparative analysis (GenBank numbers): BEV1‐PWil1 (AY028702), BEV1‐Bank10 (AY189385), BEV2‐Bank19 (AY189390), BEV5‐Mal3 (AY189398), BEV8‐Mal15 (AY189395), BEV9‐Batu20 (AY189422), BEV11‐PKW8 (AY189442), BEV12‐OBLE3 (AY189412), BEV13‐Shiz2 (AY189378), BEV14‐PKW12 (AY189436), BEV25‐Batu10 (AY189420), BEV‐NGA integrated in *Musa acuminata* subsp. *malaccensis* ‘Pahang’ (from genomic scaffold 305, HE806766)

All PCR product sequences were aligned to generate a phylogenetic tree (Figure 4). Our analysis included BEVs that are closely related to our sequences from among the different subgroups defined by Geering et al. (2005). As expected, all amplified sequences belong to clade 2 and most sequences grouped with the reference sequence of the amplified BEVs. Notably, all BEVs identified here can be assigned to a BEV subgroup described by Geering et al. (2005) (Figure 4) and exhibit >85% nucleotide sequence similarity with other sequences from the same subgroup. BEV UD is close to BEV5 with 92.7%–98.8% nucleic acid identity, BEV UH to BEV13 (94.3%–97.4% identity), BEV NGA to BEV2 (96.3%–98.3% identity), and BEV F to BEV1 (86.2%–98.8% identity). Sequences amplified by BEV UC primers were divided into two subgroups: one resembles BEV9 (90.4%–98.5% identity) and the other BEV8 (samples M24C and M27C, 89% identity). Similarly, sequences amplified by BEV UG primers are related to BEV11 (97.4%–98.8% identity) in sequences originating from *M. balbisiana* and to BEV12 (95.7%–99.1% identity) in all other sequences. Interestingly, two new sequence groups were revealed due to weak specificity of the first BEV UD and UF primer sets designed; named P and Q, respectively, both are related to known BEVs, that is, BEV25 for BEV P (93%–95.5% identity) and BEV14 for BEV Q (92% identity). New BEV P‐ and Q‐specific primers were then used to rescreen all samples. Interestingly, only *M. balbisiana* samples harbour BEVs P and Q (Table 3). As observed in the phylogenetic tree (Figure 4), BEV UF is divided into two subgroups; one is more closely linked to BEV UD (nucleotide identity c.85%), which could be derived from BEV UF.

### 2.4 Clade 2 badnaviral sequences are spread widely in the *Musa* diversity

**FIGURE 5:**
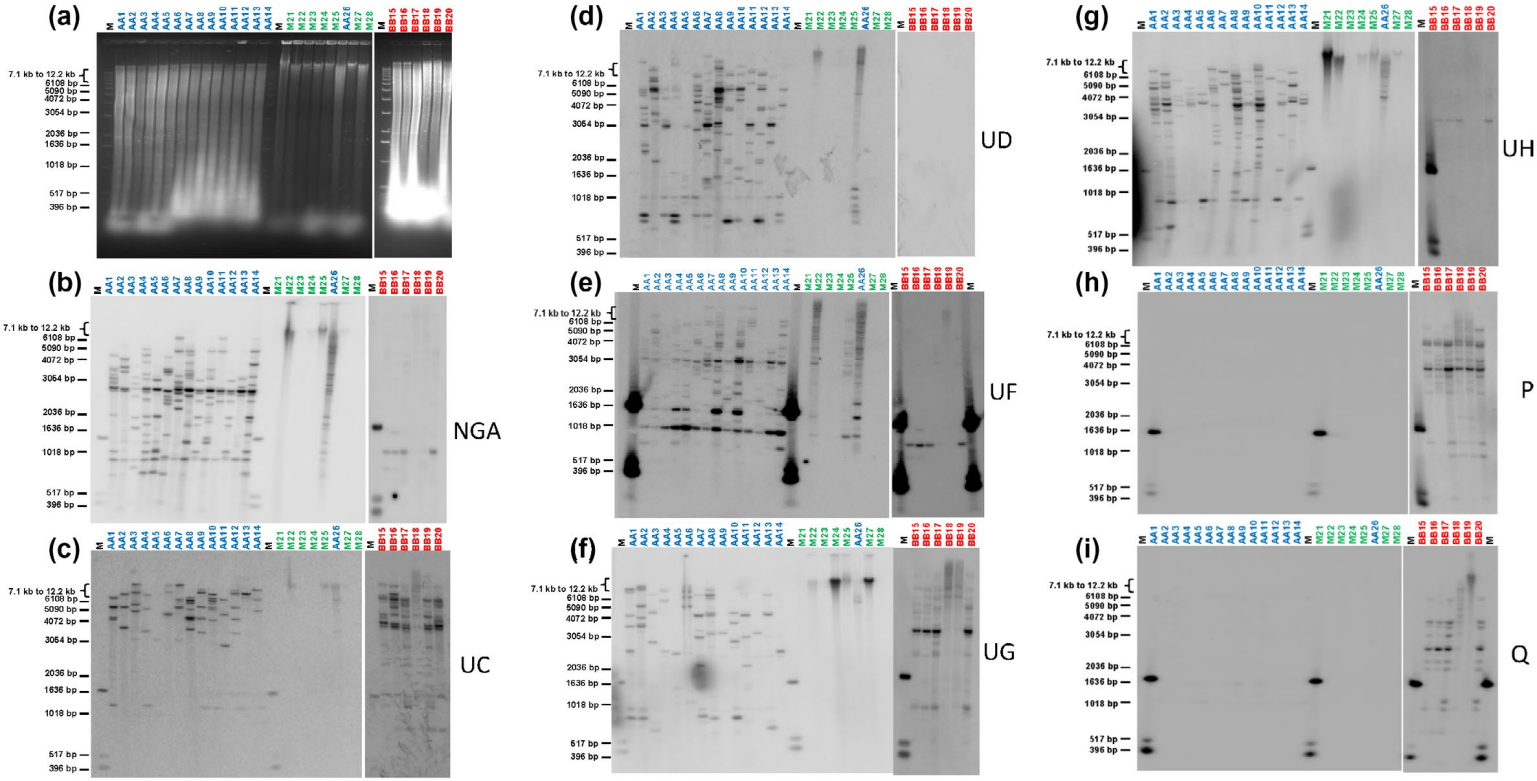
Southern blot hybridization of total genomic DNA (digested by *Hin*dIII) from plants of the family Musaceae using diverse clade 2 badnaviral sequences as probes. Samples AA1–14 and AA26 correspond to *M. acuminata* genotypes, samples BB15–20 to *M. balbisiana* genotypes, samples M21–25, M27, and M28 to other species from the family Musaceae. (a) Agarose gel electrophoresis. (b)–(i) Hybridization results for BEV probes UC (b), UD (c), UF (d), UG (e), UH (f), NGA (g), P (h), and Q (i). M, 1 kb ladder from Invitrogen

To further characterize BEVs in the *Musa* diversity, we hybridized banana genomic DNA of the same samples with probes corresponding to individual BEVs (Figure 5). Genomic DNA was digested by *Hin*dIII, which does not cleave BEVs UC, UD, UF, NGA, and P probe sequences but recognizes a single conserved site in BEVs UG, UH, and Q. No signal was observed for samples corresponding to other genera of the family Musaceae (M32 and M34) (data not shown).

Importantly, no bands corresponding to episomal viruses were observed in any of the samples. Overall, Southern blot hybridizations and PCR results were very consistent, although a few samples generated hybridization signals while the corresponding PCR was negative (e.g., samples AA4, AA5, AA14 plus AA26 with the BEV UG probe). A mismatch at the primer recognition site seems the most likely explanation. In addition, although membranes were washed stringently, we cannot rule out slight cross‐hybridization yielding a very weak signal, as explained further below. The BEV NGA probe hybridized with all *M. acuminata* samples, showing several bands ranging from high to low molecular weight, revealing several BEV NGA sequences in those samples. Although individuals displayed different patterns, they all shared two conserved fragments at 1 kb and c.3 kb (Figure 5b). In four *M. balbisiana* samples (BB15–BB17 and BB20), a similarly weak signal was seen at c.1 kb. This signal is probably due to cross‐hybridization with BEV UF because no PCR amplification was obtained with BEV NGA primers on *M. balbisiana* samples, hybridization patterns were similar to those of BEV UF but fainter, and BEV UF shares c.85% identity with BEV NGA. Geering et al. (2005) also observed the absence of BEV 2 (similar to BEV NGA) in *M. balbisiana*. We also observed hybridization signals corresponding to high molecular weight (>12 kb) bands in samples of other species of the genus *Musa* (M22, M24, M25, and M27). Similar to the NGA probe, we observed hybridization signals both for *M. acuminata* and *M. balbisiana* with BEV UC, UF, UG, and UH probes (Figure 5c,e–g). Hybridization patterns differed for all *M. acuminata* samples for each given probe and between probes, whereas hybridization patterns in *M. balbisiana* plants were much more conserved, with fewer integrations detected except for BEV UC, for which polymorphic patterns were observed (Figure 5c). Again, hybridization signals corresponding to high molecular weight (>12 kb) bands were present in some species other than *M. acuminata* and *M. balbisiana* belonging to the genus *Musa*. Finally, corroborating PCR amplification results, the three remaining probes (BEV‐UD, ‐P, and ‐Q) exhibited very striking pattern differences between *M. acuminata* and *M. balbisiana* samples, with BEV UD hybridizing only with *M. acuminata* individuals (Figure 5d), and BEV P (Figure 5h) and Q (Figure 5i) probes hybridizing only with *M. balbisiana* samples.

## 3 DISCUSSION

### 3.1 Badnaviral sequences from clade 2 are integrated in the banana genome

Previous studies differed on whether BEV UC, UD, UF, UG, and UH species corresponded to integrated and/or episomal viruses (Gayral & Iskra‐Caruana, 2009; Harper et al., 2005). To address this question, we performed DNA analysis on EAH AAA triploids collected in BSV‐affected areas in Uganda as well as on a diverse sampling encompassing BSV‐free diploids *M. acuminata* and *M. balbisiana* and other *Musa* species from the family Musaceae.

Our data revealed BEV integration in *M. acuminata* and *M. balbisiana* genomes, with hybridization signals observed for plants with symptoms (EAH AAA samples) and without symptoms (EAH AAA samples plus all the *Musa* diversity), consistent with the conclusion that BEV sequences are integrated in all our samples. For the first time, we show that some BEV sequences are shared between *M. acuminata* and *M. balbisiana* genomes.

Our Southern blot results (Figure 5) indicate the level of plant genome colonization, and suggest coevolution between virus and banana. All *M. acuminata* banana samples showed different integration patterns, suggesting a large degree of badnavirus sequence colonization via either multiple integration waves or genome duplications and evolution of one or a few initial integration events. Interestingly, each given probe suggested a wide polymorphism of badnavirus sequence integration in all *M. acuminata* banana samples, probably reflecting genetic and geographical *M. acuminata* subspecies diversity linked to various environmental pressures (Perrier et al., 2011). Our data are consistent with preliminary analysis by D’Hont et al. (2012), who reported the presence of different BEVs belonging to clade 2 at several loci distributed on 10/11 chromosomes of the genome of *M. acuminata* ‘Pahang’.

The patterns observed for *M. balbisiana* are less complex, suggesting either fewer integrations or very few genome duplication events with a much less diverse profile. This low polymorphism of badnavirus sequence integration could be due to limited genetic diversity among *M. balbisiana* banana species (Carreel, 1994; Gayral et al., 2010). Overall, our data corroborate preliminary data published by Geering et al. (2001), who reported integrations in all samples, but fewer in *M. balbisiana* than in *M. acuminata*. Importantly, we demonstrate here for the first time that several BEVs are integrated in other species of the genus *Musa*, that is, *M. basjoo*, *M. itinerans*, *M. laterita*, *M. ornata*, and *M. schizocarpa,* (sections *Eumusa* or *Rhodochlamys*), but not in more phylogenetically distant samples corresponding to *Callimusa* and *Australimusa* sections or other genera of the family Musaceae (Table 3 and data not shown). This suggests that, for some BEVs, initial integrations occurred after formation of the family Musaceae but before speciation of the genus *Musa* (see below).

BEVs UC and UG appear to be widely disseminated within *M. acuminata* and *M. balbisiana*, unlike BEV UF, which is abundant in *M. acuminata* but poorly represented in *M. balbisiana*. Our results also show the absence of BEVs UD, UH, and NGA in *M. balbisiana* genomes, the absence of BEVs P and Q in *M. acuminata* genomes and the presence of all BEVs (except P and Q) in some other species of the family Musaceae.

Thus, our results differ from those of Harper et al. (2005), who suggested that BEVs UC, UD, UF, UG, and UH exist as episomal BSV particles, and support the hypothesis proposed by Gayral and Iskra‐Caruana (2009), ascribing an exclusively integrated status to all BEVs of clade 2. Our present results allow us to ascribe the diversity of BSV exclusively to clades 1 (seven species to date: BSOLV, BSGFV, BSMYV, BSIMV, BSCAV, BSUAV, and BSVNV) and 3 (five species to date: BSUIV, BSULV, BSUMV, BSUJV, and BSUKV).

### 3.2 What can BEVs tell us about the badnavirus/banana coevolution?

Badnaviral sequences linked to banana plants are distributed over three main clades (Figure 4). Surprisingly, they are as diverse as all the other viruses of the genus *Badnavirus* with which they share a same common ancestor. Interestingly, the clade to which these sequences belong is associated with a particular status (episomal and/or integrated) as a result of specific interactions between the virus and its banana host.

Clade 1 encompasses BSVs that are either episomal sensu stricto or both episomal and integrated (Iskra‐Caruana et al., 2014) as observed for BSOLV, BSGFV, BSIMV, and BSMYV, where integrations restricted to *M. balbisiana* genomes occurred only after speciation of *M. acuminata/M. balbisiana*, but before diversification of *M. balbisiana* (Chabannes et al., 2013). Although those eBSV exhibit a strong rearranged structure, with inverted and duplicated sequences attesting to past integration, pseudogenization has not progressed to the point where they can no longer reconstitute an infectious viral genome (Chabannes et al., 2013; Chabannes & Iskra‐Caruana, 2013; Iskra‐Caruana et al., 2010). Because nucleic acid identity between a given eBSV and the corresponding BSV is >99%, it is likely that episomal BSOLV, BSGFV, and BSIMV observed now are due mainly, or exclusively, to the awakening of an endogenous counterpart (Chabannes et al., 2013; Gayral et al., 2008).

Because no episomal virus belonging to clade 2 has ever been detected in banana plants, we assume that the episomal BEV counterpart has long since disappeared. BEVs are thus relics of ancestor viruses that existed previously in wild plant populations, before speciation of the genus *Musa* and long before domestication and trade. Consequently, they are older than the BSVs and eBSV of clade 1, that is, the BSOLV, BSMYV, BSIMV, and BSGFV integrated in *M. balbisiana* genomes, with integration estimated to have occurred c.640,000 years ago (Gayral et al., 2010). Whether integrated BEV sequences confer a selective advantage by contributing towards plant virus resistance (via transcriptional or posttranscriptional gene silencing) is still an open question, but would explain both the disappearance of the counterpart episomal viruses and current integrated sequences resulting from pseudogenization. As described for other viruses (Feschotte & Gilbert, 2012), BEVs have become part of the genetic material of the banana host.

BSVs of clade 3 result from recent viral evolution in East Africa. Karamura et al. (1996) described a widespread BSV epidemic in Uganda on the East African Highland *M. acuminata* banana group (EAH AAA) (Kubiriba et al., 1997). The endemic presence of BSV in Uganda is probably the result of vegetative propagation of infected plants rather than vector transmission because cultivated cultivars are seedless and the rate of disease spread by mealybugs is slow (Daniells et al., 2001b; Kubiriba et al., 2001). Furthermore, BSVs generally do not have a severe impact on bunch production, particularly when cultural conditions are good. In the 109 EAH AAA samples analysed here, we observed a prevalence of species BSUIV, BSULV, and BSUMV from clade 3 (Table 2), whereas species BSUJV and BSUKV are poorly represented or absent, respectively, in agreement with the findings of Harper et al. (2005) (after correction of BSV species names mislabelled in the latter article; the species BSUHV, BSUIV, BSUJV, BSUKV, and BSULV described by Harper et al., 2005, were respectively submitted to GenBank as BSUIV, BSUJV, BSUKV, BSULV, and BSUMV). Importantly, no sequences corresponding to BSUIV, BSULV, and BSUMV have so far been found integrated in banana plants (Figure 2a). The diversity of viruses from clade 3 observed nowadays is therefore more likely due either to introduction of infected banana cultivars from different locations or to host shifts between banana and other plants (Iskra‐Caruana et al., 2014).

### 3.3 What can BEVs tell us about the banana phylogeny?

**FIGURE 6:**
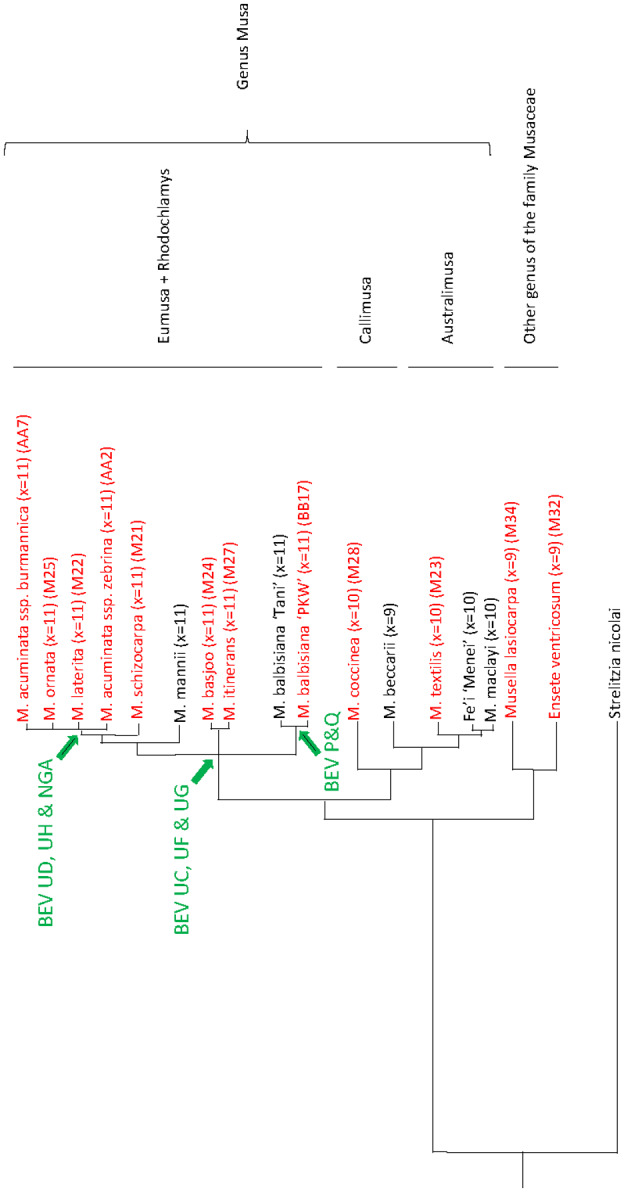
Schematic phylogenetic tree of BEV fixation events relative to speciation events within the family Musaceae constructed using nodes supported from the latest *Musa* phylogenies (Christelova et al., 2011; Janssens et al., 2016; Li et al., 2010). Banana samples from different sections of the genus *Musa* and other genera of the family Musaceae are represented. Samples characterized during this work are in red. *M. acuminata* and *M. balbisiana* subspecies are represented by samples AA2 and AA7, and BB17, respectively. Green arrows, BEV fixation events; X, number of chromosomes

BEVs phylogeny suggests two close (in terms of parsimony) scenarios of banana genome integration: (a) a unique integration event before emergence of Musaceae, associated with several gene duplications and losses in some *Musa* subgroups; and (b) multiple integrations, whereby badnavirus ancestors of BEVs integrated into the banana genome in at least three waves corresponding to distinct periods of banana evolution, as proposed by Yu et al. (2019) for other endogenous pararetroviruses in Citrinae. Synteny analyses between the *M. acuminata* Pahang (D’Hont et al., 2012) and PKW (Wang et al., 2019) genomes found no evidence that any BEV‐containing loci were identical. This is not too surprising considering that the number of initial integrations is low, and that only half of the genomes of these two sequenced plants are being examined, because each derives from a duplication of an initial haploid plant. On the other hand, because genomes A and B diverged 4.5 million years ago (Lescot et al., 2008), the initial integration loci may have diverged sufficiently so as to no longer be identified during synteny analyses. Therefore, based on recently published banana phylogenies (Christelova et al., 2011; Janssens et al., 2016; Li et al., 2010), we propose a speculative scheme (Figure 6) depicting each BEV integration event for scenario (b) with regards to the main speciation events reported within the family Musaceae.

**FIGURE 7:**
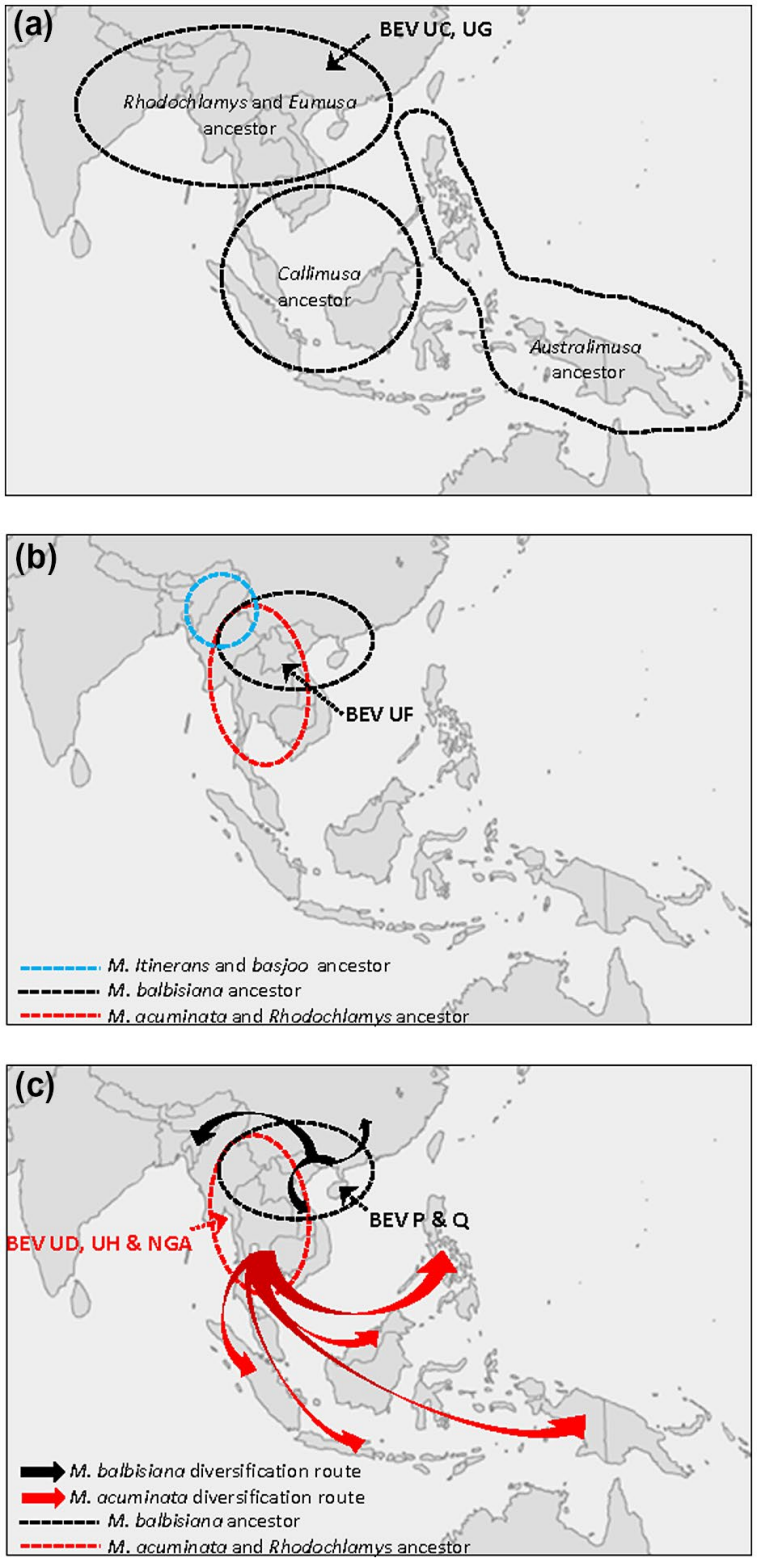
Geographical distribution of main sections of the genus *Musa* and subspecies *Eumusa* along with proposed BEV fixation events. A first wave of BEV fixation (BEV UC and UG) occurred in the *Rhodochlamys* and *Eumusa* ancestor (a). A second wave of integration fixed BEV UF in the *M. balbisiana*/*M. acuminata*/*Rhodochlamys* ancestor but not in the *M. itinerans/M. basjoo* ancestor (b). The boundaries of the *M. itinerans*/*M. basjoo* ancestor are uncertain. Finally, a third wave of BEV fixation occurred in *M. acuminata* (BEV UD, UH, and NGA) and *M. balbisiana* (P and Q) ancestors prior to diversification of both subspecies (c). *M. balbisiana* and *M. acuminata* diversification routes are illustrated by black and red arrows, respectively. All BEV integration and fixation events proposed took place in South/South‐East continental Asia. The figure was inspired by Simmonds (1962), De Langhe et al. (2009), and Perrier et al. (2011)

The presence of BEVs UC, UF, and UG within both *M. acuminata* and *M. balbisiana* genomes, as well as other species of the genus *Musa* (*M. basjoo*, *M. itinerans*, *M. laterita*, or *M. ornata*), could represent the first and second integration waves (Figure 7a,b). Indeed, BEV UF integration may have followed that of BEV UC and BEV UG because *M. basjoo* and *M. itinerans* genomes do not harbour BEV UF integrations (Table 3). Interestingly, amongst *Musa* sections, BEVs are present only in *Rhodochlamys* (*M. laterita* and *M. ornata*) and *Eumusa* (*M. acuminata*, *M. balbisiana*, *M. schizocarpa*, *M. basjoo*, and *M. itinerans*) and are absent from both *Australimusa* (*M. textilis*) and *Callimusa* (*M. coccinea*)—phylogenetically very distant from *Rhodochlamys* and *Eumusa* (Christelova et al., 2011; Li et al., 2010). According to estimates of species divergence times within the family Musaceae (Christelova et al., 2011), integrations of BEVs UC, UF, UG took place between c.28 and 50 million years ago, corresponding to speciation events within *Rhodochlamys*/*Eumusa* and the age of the genus *Musa*, respectively. Importantly, based on the known geographical distribution of the main sections of the genus *Musa* (De Langhe et al., 2009) and the *M. acuminata* and *M. balbisiana* subspecies (Perrier et al., 2011), the absence of BEV in *Australimusa* and *Callimusa* genomes, and their presence in all *M. acuminata* and *M. balbisiana* subspecies, indicates that initial badnavirus infections of the *Musa* ancestor and viral integration of BEVs UC, UF, and UG occurred in South/South‐East continental Asia (Figure 7a). Using the BEV markers developed here, we can further restrict the origin of the *Eumusa*/*Rhodochlamys* ancestor to the south mainland of Asia, thus narrowing considerably the area defined initially by De Langhe et al. (2009).

Alternatively, virus ancestors of BEVs UC, UF, and UG could have integrated independently and randomly into different *Musa* genomes. This hypothesis is less parsimonious because it requires that at least three distinct viruses (BEVs UC, UF, and UG) integrated into different *Musa* genomes over time and in different geographical areas.

The presence of BEVs UD, UH, and NGA only in the genome of *M. acuminata*, in contrast to the absence of BEVs P and Q (present only in *M. balbisiana*), suggests a third wave of integrations occurring after *M. acuminata* and *M. balbisiana* speciation (Figures 6 and 7c) in scenario (b). Considering the close phylogenetic relationship of BEVs UF and UD (Figure 4), we propose that BEV UD emerged after a duplication of BEV UF in *M. acuminata* only. Interestingly, Southern blot hybridization patterns clearly indicate that this integration occurred after speciation of *M. acuminata*/*M. balbisiana* but before species diversification. This third wave of integration seems likely to have been a host response to different viral pressures, namely BEVs P and Q for *M. balbisiana* and BEVs NGA, UD, and UH for *M. acuminata*, which come from different geographical areas (Perrier et al., 2011) (Figure 7b,c). Interestingly, within the genus *Musa*, PCR and Southern blot profiles of BEV species suggest a close relationship between *M. acuminata* subspecies and banana species from section *Rhodochlamys* (*M. ornata* M25 and *M. laterita* M22), in agreement with published *Musa* phylogeny (Christelova et al., 2011; Li et al., 2010). Similarly, profiles of *M. basjoo* (M24) and *M. itinerans* (M27) are barely distinguishable, again corroborating their closeness in *Musa* phylogeny (Li et al., 2010). Notably, both exhibit intermediate BEV profiles compared with *M. acuminata* and *M. balbisiana*, supporting their equidistant position from the latter in the phylogenetic tree of Musaceae (Li et al., 2010).

Duroy et al. (2016) previously demonstrated eBSV to be relevant phylogenetic markers to illustrate the *M. balbisiana* phylogeographic story. In light of our data, and given the high polymorphism of BEVs within *M. acuminata* species, BEV patterns can be added to the arsenal of phylogenetic markers to describe and complete *Musa* phylogeny.

## 4 EXPERIMENTAL PROCEDURES

### 4.1 Plant material

Leaf samples from 109 East African Highland banana plants representing five clone sets encompassing EAH AAA banana genetic diversity (Nakitembe [32], Musakala [19], Nakabulu [16], Nfuuka [23], and Nbide [beer cultivar, 19]; Karamura & Pickersgill, 1999) were collected in Uganda in 2009; 91 samples showed typical banana leaf streak mosaic symptoms, indicating BSV infection.

We also analysed 30 samples encompassing different *M. acuminata* (AA1–AA14, AA26) and *M. balbisiana* (BB15–BB20) diploid subspecies, along with other species from the genus *Musa* (M21–M25, M27, and M28) and other genera of the family Musaceae (M32 and M34) (Table 4). Samples were collected in the Banana field collection of CIRAD Neufchateau, Guadeloupe, France.

**TABLE 4:** Description of plant material used in this study

Genus or section in the family Musaceae	Species/subspecies	Common name	Genome	Accession number	Number in that study
*Eumusa*	*M. acuminata* subsp. *errans*	Agutay	AAw	ITC1028 (NEU0033)	AA1
*Eumusa*	*M. acuminata* subsp. *zebrina*	Zebrina	AAw	ITC1177 (NEU0029)	AA2
*Eumusa*	*M. acuminata* subsp. *siamea*	Pa (Rayong)	AAw	ITC0672 (NEU0024)	AA3
*Eumusa*	*M. acuminata* subsp. *malaccensis*	Pahang	AAw	ITC0609 (NEU0013)	AA4
*Eumusa*	*M. acuminata* subsp. *malaccensis*	THA018	AAw	ITC1067 (NEU0034)	AA5
*Eumusa*	*M. acuminata* subsp. *truncata*	Truncata	AAw	(NEU0027)	AA6
*Eumusa*	*M. acuminata* subsp. *burmannica*	Long Tavoy	AAw	ITC0283 (NEU0016)	AA7
*Eumusa*	*M. acuminata* subsp. *microcarpa*	Bornéo	AAw	ITC0253 (NEU0028)	AA8
*Eumusa*	*M. acuminata* subsp. *malaccensis*	Malaccensis	AAw	ITC0250	AA9
*Eumusa*	*M. acuminata*	Wikago	AAcv	NEU0102 (ITC0888)	AA10
*Eumusa*	*M. acuminata* subsp. *siamea*	Khae (Phrae)	AAw	ITC0660 (NEU0025)	AA11
*Eumusa*	*M. acuminata* subsp. *malaccensis*		AAw	ITC0399	AA12
*Eumusa*	*M. acuminata* subsp. *burmannicoides*	Calcutta 4	AAw	ITC0249 (NEU0017)	AA13
*Eumusa*	*M. acuminata* subsp. *siamea*	Pa (Songkhla)	AAw	ITC0408 (NEU0043)	AA14
*Eumusa*	*M. acuminata* subsp. *banksii*	Madang	AAw	ITC0254	AA26
*Eumusa*	*M. balbisiana*	Honduras	BBw	ITC0247 (NEU0049)	BB15
*Eumusa*	*M. balbisiana*	Cameroun	BBw	ITC0246 (NEU0050)	BB16
*Eumusa*	*M. balbisiana*	Pisang Klutuk Wulung	BBw	ITC1063 (NEU0056)	BB17
*Eumusa*	*M. balbisiana*	Butuhan	BBw	ITC0565	BB18
*Eumusa*	*M. balbisiana*	Lal Velchi	BBw	NEU0051	BB19
*Eumusa*	*M. balbisiana*	Papouasie New Guinea	BBw	ITC0626	BB20
*Eumusa*	*M*. *schizocarpa*		SS	ITC856	M21
*Rhodochlamys*	*M. laterita*			NEU0008	M22
*Australimusa*	*M. textilis*		TT	ITC1072 (NEU0001)	M23
*Eumusa*	*M. basjoo*			NEU0060	M24
*Rhodochlamys*	*M. ornata*			NEU0007	M25
*Eumusa*	*M. itinerans*				M27
*Callimusa*	*M. coccinea*			ITC0287 (NEU0003)	M28
*Ensete*	*Ensete ventricosum*				M32
*Musella*	*Musella lasiocarpa*				M34

*Note*. ITC numbers, accession codes from the International Transit Center, Catholic University, Leuven, Belgium; NEU numbers, accession codes from the Banana collection of CIRAD Neufchateau, Guadeloupe, France; AAw or AAcv, wild or cultivar of diploid *acuminata* bananas; BBw, wild diploid *balbisiana* bananas; *M*, other species from the family Musaceae. Sections *Eumusa*, *Rodochlamys*, *Callimusa*, and *Australimusa* correspond to subsections in the genus *Musa*.

### 4.2 Extraction of genomic DNA from banana

Genomic DNA was extracted from fresh or frozen banana leaf tissue using the method of Gawel and Jarret (1991).

### 4.3 Immunocapture PCR detection

BSV species were detected by IC‐PCR according to a procedure adapted from Le Provost et al. (2006) using specific BSOLV, BSGFV, BSIMV, and BSMYV primers (Table 1) and a polyclonal antiserum raised against a cocktail of purified BSV species and SCBV species (kindly provided by B.E.L. Lockhart). To avoid contamination by plant genomic DNA, samples were treated with RNase‐free DNase I (Promega). DNase mix (3 µl of 10 × buffer [400 mM Tris.HCl pH 8, 100 mM MgSO_4_, 10 mM CaCl_2_], 3 µl of DNase I [1 U/µl], and 24 µl of water) was added to coated tubes and incubated for 1 hr at 37 °C. The supernatant was removed and the tubes washed once with water. DNase I was inactivated by incubation at 95 °C for 10 min.

### 4.4 PCR, cloning, and sequencing

Primers in the RTase/RNase H region of badnavirus ORF III were designed to specifically detect each Ugandan badnaviral species of clades 2 and 3 (Table 1). PCR was performed with 1 U GoTaq DNA polymerase according to the manufacturer's instructions (Promega) and the following thermal cycling conditions: 1 cycle at 94 °C for 4 min; 35 cycles at 94 °C for 30 s, Tm (Table 1) for 30 s, 72 °C for 30 s; followed by 1 cycle at 72 °C for 10 min. PCR fragments were sequenced by Beckman Coulter Genomics (UK).

PCR products used as probes for Southern blot hybridization were gel‐purified using the Wizard SV Gel and PCR Clean‐up System (Promega) and cloned into the pGEM‐T Easy vector according to the manufacturer's instructions (Promega).

### 4.5 Southern blot hybridization

Total genomic DNA (40 µg per sample) was digested overnight with 1 U/μg DNA for each enzyme in a final volume of 200 µl; *Hin*dIII for samples representative of the *Musa* diversity, and *Kpn*I or *Alw*441 for EAH banana samples. Samples were separated by electrophoresis on 1% agarose gels and capillary‐transferred overnight to Hybond N+ membrane (Amersham Biosciences) in 20 × saline‐sodium citrate (SSC) buffer. Nucleic acids were fixed onto the membrane using a UV crosslinker (70,000 μJ/cm^2^), then prehybridized in 20 ml of buffer (50 mM Tris.HCl pH 8, 25 mM ETDA, 5 × SSC, 1% sodium dodecyl sulphate [SDS], 2.5 × Denhardt's solution and 2 mg denatured salmon sperm DNA) and incubated for 3 hr at 65 °C.

Fragments corresponding to RTase/RNaseH regions of each species of clade 3 (BSUIV, BSULV, BSUMV) and each group of clade 2 (BEVs UC, UD, UF, UG, UH, P, Q, NGA) were released from pGEM‐T Easy plasmids using *Eco*RI digestion and used as probes. DNA probe (50 ng) was labelled with [α‐^32^P] dCTP using a random priming protocol (Prime‐a‐Gene kit, Promega). Labelled probe was then added to 20 ml of hybridization solution (as above + 5% dextran sulphate) and incubated overnight at 65 °C. To remove nonspecific hybridization signal, membranes were washed at 65 °C for 10 min, twice in SSC with 0.1% SDS solution and once in 0.5 × SSC with 0.1% SDS. Additional washes, if required, used 0.2 × SSC with 0.1% SDS solution. Membranes were wrapped in transparent plastic (Scel O Frais) and screen scanned after overnight exposure on a filmless autoradiography Typhoon FLA 9000 imaging system (GE Healthcare).

### 4.6 Rolling circle amplification

DNA was amplified using a TempliPhi Amplification kit (GE Healthcare) following the protocol described by James et al. (2011). Reaction products were digested using 2 U of different restriction endonucleases (Promega), according to the manufacturer's instructions, and then separated by electrophoresis in 1% agarose gels.

### 4.7 Phylogenetic analysis

Badnaviral sequences were aligned using the MAFFT software algorithm (Katoh & Standley, 2013). Phylogenetic trees were constructed using the maximum‐likelihood method with PhyML 3.0 (Guindon et al., 2010) and visualized using Darwin 5 software (Perrier et al., 2003). The robustness of trees was tested with aLRT‐SH‐like statistical support (Anisimova et al., 2011). The new sequences produced during this work have GenBank accession numbers KJ720037–KJ720154 and KJ734678–KJ734703.

## Supporting information


**FIGURE S1** Southern blot hybridization of genomic DNA from EAH AAA banana plants using clade 3 (BSUIV; a) and clade 2 (BSUFV; b) badnaviral sequences as probes. Total genomic DNA (undigested, *Kpn*I‐digested, *Alw*441‐digested): 1, Pisang Klutuk Wulung (PKW) (BB genotype); 2, Cavendish (CAV) (AAA genotype); 3, Pahang (AA genotype); 4–9, EAH AAA plants [4–8 healthy samples (symptomless and no bacilliform particles), 9 BSUIV‐infected]. M, 1 kb ladder from Invitrogen

**FIGURE S2** Southern blot hybridization of genomic DNA from EAH AAA banana plants using clade 3 (BSULV) badnaviral sequences as probes. Total genomic DNA (undigested, *Kpn*I‐digested, *Alw*441‐digested): 1, Pisang Klutuk Wulung (PKW) (BB genotype); 2, Cavendish (CAV) (AAA genotype); 3, Pahang (AA genotype); 4–9, EAH AAA plants [4, 6, 9, healthy samples (symptomless and no bacilliform particles); 5, BSUIV‐infected; 7, BSUMV‐infected; 8, BSUIV and BSULV coinfected]. M, 1 kb ladder from Invitrogen

**FIGURE S3** Bacilliform particles visualized by immunosorbent electron microscopy (ISEM) on samples with symptoms containing sequences of species from clade 3. Partially purified virus preparations from leaf samples were prepared essentially according to the method of Bouhida et al. (1993). Carbon‐coated electron microscope grids were coated with a mix of polyclonal antisera to banana streak viruses (PMX2RC supplied by BEL Lockhart, University of Minnesota) at a dilution of 5 µg/ml in 60 mM sodium carbonate buffer, pH 9.5, for 30 min at room temperature. The grids were then washed twice for 5 min with the same buffer and incubated with 10 μl of partially purified virus preparation at room temperature for 2 hr. After rinsing with water, the grids were stained with 4% uranyl acetate or 2% (wt/vol) potassium phosphotungstate pH 7.0 and viewed under a 100 CX II transmission electron microscope (Jeol) (Bouhida et al., 1993, An analysis of the complete sequence of a sugarcane bacilliform virus genome infectious to banana and rice. *Journal of General Virology*, 74, 15–22)

## Data Availability

The data that support the findings of this study are openly available in NCBI GenBank at https://www.ncbi.nlm.nih.gov/nucleotide, reference numbers KJ720037–KJ720154 and KJ734678–KJ734703.
