# Supplementary figures and images for: Badnaviruses and banana genomes: a long association sheds light on Musa phylogeny and origin
Source: Mol Plant Pathol. 2020 Nov 24;22(2):216–30. doi: 10.1111/mpp.13019 (PMC7814968; doi:10.1111/mpp.13019)

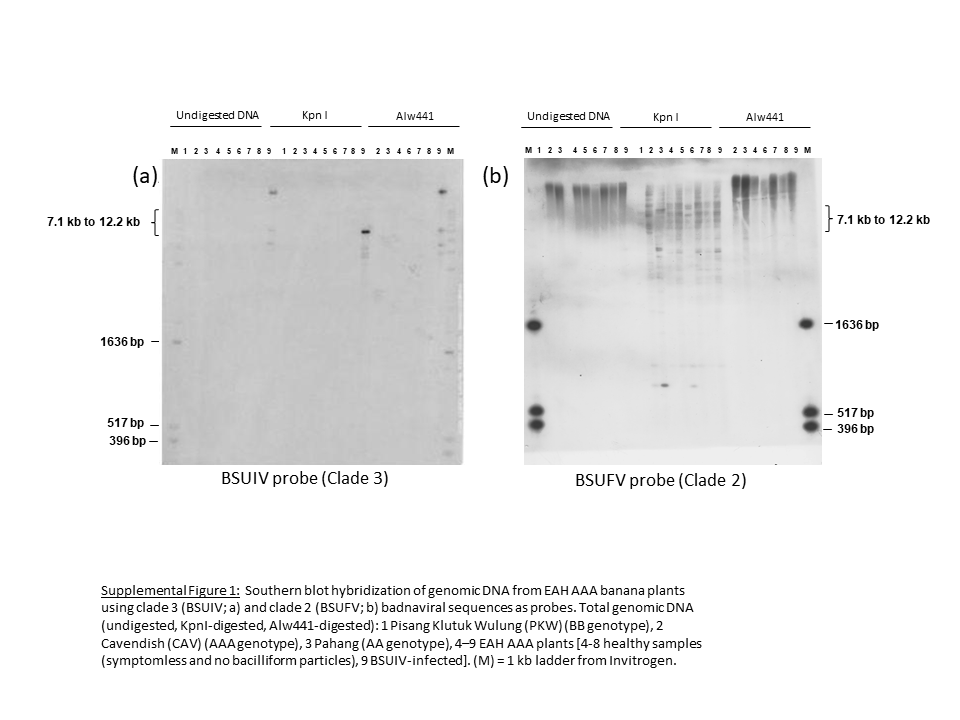

Supplement: Supplementary file 1 — FIGURE S1 Southern blot hybridization of genomic DNA from EAH AAA banana plants using clade 3 (BSUIV; a) and clade 2 (BSUFV; b) badnaviral sequences as probes. Total genomic DNA (undigested, KpnI‐digested, Alw441‐digested): 1, Pisang Klutuk Wulung (PKW) (BB genotype); 2, Cavendish (CAV) (AAA genotype); 3, Pahang (AA genotype); 4–9, EAH AAA plants [4–8 healthy samples (symptomless and no bacilliform particles), 9 BSUIV‐infected]. M, 1 kb ladder from Invitrogen [file MPP-22-216-s001.tif]

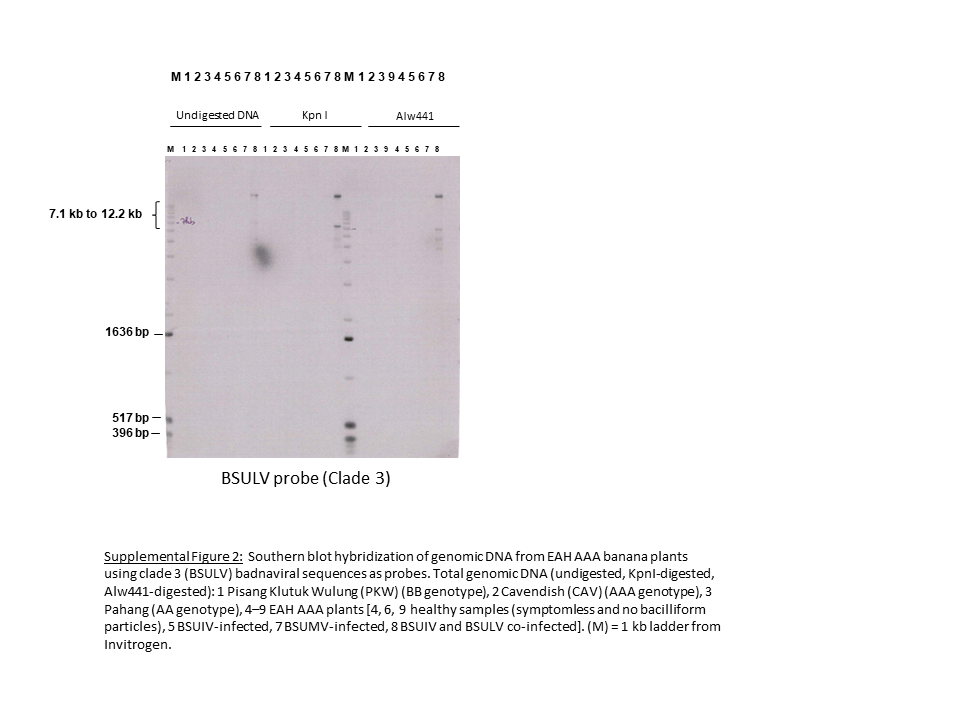

Supplement: Supplementary file 2 — FIGURE S2 Southern blot hybridization of genomic DNA from EAH AAA banana plants using clade 3 (BSULV) badnaviral sequences as probes. Total genomic DNA (undigested, KpnI‐digested, Alw441‐digested): 1, Pisang Klutuk Wulung (PKW) (BB genotype); 2, Cavendish (CAV) (AAA genotype); 3, Pahang (AA genotype); 4–9, EAH AAA plants [4, 6, 9, healthy samples (symptomless and no bacilliform particles); 5, BSUIV‐infected; 7, BSUMV‐infected; 8, BSUIV and BSULV coinfected]. M, 1 kb ladder from Invitrogen [file MPP-22-216-s002.tif]
