# Supplementary material for: Badnaviruses and banana genomes: a long association sheds light on Musa phylogeny and origin
Source: Mol Plant Pathol. 2020 Nov 24;22(2):216–30. doi: 10.1111/mpp.13019 (PMC7814968; doi:10.1111/mpp.13019)
Supplement: Supplementary file 3 — FIGURE S3 Bacilliform particles visualized by immunosorbent electron microscopy (ISEM) on samples with symptoms containing sequences of species from clade 3. Partially purified virus preparations from leaf samples were prepared essentially according to the method of Bouhida et al. (1993). Carbon‐coated electron microscope grids were coated with a mix of polyclonal antisera to banana streak viruses (PMX2RC supplied by BEL Lockhart, University of Minnesota) at a dilution of 5 µg/ml in 60 mM sodium carbonate buffer, pH 9.5, for 30 min at room temperature. The grids were then washed twice for 5 min with the same buffer and incubated with 10 μl of partially purified virus preparation at room temperature for 2 hr. After rinsing with water, the grids were stained with 4% uranyl acetate or 2% (wt/vol) potassium phosphotungstate pH 7.0 and viewed under a 100 CX II transmission electron microscope (Jeol) (Bouhida et al., 1993, An analysis of the complete sequence of a sugarcane bacilliform virus genome infectious to banana and rice. Journal of General Virology, 74, 15–22) [file MPP-22-216-s003.docx]

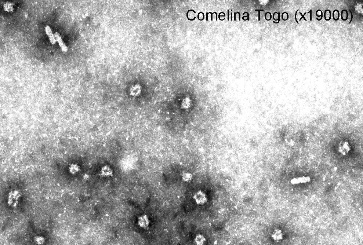


Supplemental Figure 3 : Bacilliform particles visualized by Immunosorbent electron microscopy (ISEM) on symptomatic samples containing sequences of species from clade 3.

Partially purified virus preparations from leaf samples were prepared essentially according to the method of (Bouhida et al., 1993). Carbon-coated electron microscope grids were coated with a mix of polyclonal antisera to Banana streak viruses (PMX2RC supplied by BEL Lockhart, University of Minnesota) at a dilution of 5 µg/ml in 60 mM sodium carbonate buffer, pH 9.5, for 30 min at room temperature. The grids were then washed twice for 5 min with the same buffer and incubated with 10 μL of partially purified virus preparation at room temperature for 2 h. After rinsing with water, the grids were stained with 4% uranyl acetate, or 2 % (w/v) potassium phosphotungstate pH 7.0 and viewed under a 100 CX II transmission electron microscope (Jeol).

Bouhida, M., Lockhart, B. E. and Olszewski, N. E. (1993) An analysis of the complete sequence of a sugarcane bacilliform virus genome infectious to banana and rice. *J Gen Virol,* **74,** 15-22.
